# Supplementary material for: Perturbation biology nominates upstream–downstream drug combinations in RAF inhibitor resistant melanoma cells
Source: eLife. 2015 Aug 18;4:e04640. doi: 10.7554/eLife.04640 (PMC4539601; doi:10.7554/eLife.04640)
Supplement: Supplementary file 1. — (A) Drugs used in perturbation experiments. (B) Proteins that respond to at least one perturbation and exist in models. (C) Perturbation conditions. (D) Predicted G1-arrest response to combined targeting of c-Myc and other nodes (Perturbations on c-Myc + X). (E) Statistical validation of perturbation models. (F) Predicted co-targeting strategies in SkMel133 cell line. DOI: http://dx.doi.org/10.7554/eLife.04640.029 [file elife04640s001.docx]

**Supplementary Material**

**Supplementary Tables**

Supplementary File 1A- Drugs used in perturbation experiments

Supplementary File 1B- Proteins that respond to at least one perturbation and exist in models

Supplementary File 1C- Perturbation conditions

Supplementary File 1D- Predicted G1-arrest response to combined targeting of c-Myc and other nodes (Perturbations on c-Myc + X).

Supplementary File 1E- Statistical validation of perturbation models

Supplementary File 1F- Predicted co-targeting strategies in SkMel133 cell line

**Table S1A**. Drugs used in perturbation experiments

| Drug name | Target | Downstream effector | Dose 1 (nM) | Dose 2 (nM) |
| --- | --- | --- | --- | --- |
| AKTi 1/2 | AKT | AKTpS473 | 5.000 | 10.000 |
| Temsirolimus | mTOR | S6pS240 | 0.3 | 0.6 |
| ZSTK474 | PI3K | AKTpS473 | 600 | 1200 |
| PD0325901 | MEK1/2 | MAPK1pT202 | 1.5 | 3 |
| Stattic | STAT3 | STAT3pY705 | 600 | 1200 |
| HNHA | HDAC | P27/Kip1 | 6,000 | 12,000 |
| PLX4720 | BRAF^V600E^ | MAPK1pT202 | 60 | 120 |
| RO-31-7549 | PKC | S6pS240 | 3500 | 7000 |
| RY | CDK4 | Rb_pS807 | 2000 | NA |
| P6 | JAK | GSK3pS21 | 20 | 40 |
| SR | SRC | GSK3pS21 | 2,400 | 4,800 |
| Nutlin | MDM2 | 4EBP1pS65 | 3,000 | 6,000 |

**Table S1B.** Proteins that respond to at least one perturbation and exist in models

| Protein | Phosphorylation |  |  | Protein | Phosphorylation |
| --- | --- | --- | --- | --- | --- |
| 4EBP1 | S65 |  |  | IGF1Rβ | |
| 4EBP1 | T37 |  |  | IGFBP2 | |
| 4EBP1 | T70 |  |  | IRS1 | S307 |
| α -Tubulin | |  |  | IRS1 | |
| ACC1 | S79 |  |  | ERK/MAPK1 | T202 |
| ACC1 |  |  |  | MEK | S217 |
| AKT | S473 | |  | mTOR | S2448 |
| AKT | T308 | |  | p21/Cip1 |  |
| AKT |  |  |  | p27/Kip1 |  |
| AMPK | T172 | |  | p38/MAPK14 | T180 |
| ATM | S1981 | |  | p38/MAPK14 | |
| ATR |  |  |  | P53 |  |
| β-Catenin | S33 |  |  | p70S6K | T389 |
| β-Catenin | |  |  | PAI-1 | |
| BAK |  |  |  | PAX2 | |
| BCL-XL |  |  |  | PCNA | |
| BCL2 |  |  |  | PDK1 | S241 |
| BIM |  |  |  | PI3Kp85 |  |
| BRAF |  |  |  | PKCa | |
| c-JUN | S73 |  |  | PLK1 | |
| c-Myc |  |  |  | RAD51 | |
| Caspase9 | |  |  | Rb | S807 |
| Caspase9clvdAsp31 |  |  |  | S6 | S235 |
| Caveolin | |  |  | S6 | S240 |
| CHK1 | S345 | |  | S6 | |
| CHK2 | T68 | |  | SMAD3 | S423 |
| Collagenase | |  |  | SMAD3 | |
| COX2 |  |  |  | SRC | Y527 |
| cRAF | |  |  | SRC | Y416 |
| CyclinB1 | |  |  | SRC |  |
| CyclinD1 | |  |  | STAT3 | Y705 |
| CyclinE1 | |  |  | STAT3 | |
| EGFR | |  |  | STAT5 | Y694 |
| ELK1 | S383 | |  | STAT5 | |
| ER-α |  |  |  | STAT6 | Y641 |
| Fibronectin | |  |  | TAZ | S89 |
| GATA3 | |  |  | TSC2 | T1462 |
| GSK3-αβ | S21 |  |  | TSC2 | |
| GSK3-αβ | S9 |  |  | XRCC1 | |
| GSK3-αβ | |  |  | YAP | S127 |
| HSP27 |  |  |  | YBI | S102 |

**Table S1C.** Perturbation conditions

| Index | Perturbation1 | Dose1 (nM) | Perturbation2 | Dose2  (nM) | Index | Perturbation1 | Dose1  (nM) | Perturbation2 | Dose2  (nM) |
| --- | --- | --- | --- | --- | --- | --- | --- | --- | --- |
| 1 | MEKi | 1.5 |  |  | **46** | BRAFi | 60 | MDM2i | 3000 |
| 2 | MEKi | 1.5 | HDACi | 6,000 | **47** | BRAFi | 60 | JAKi | 20 |
| 3 | MEKi | 1.5 | MDM2i | 3,000 | **48** | BRAFi | 60 | PKCi | 3500 |
| 4 | MEKi | 1.5 | JAKi | 20 | **49** | BRAFi | 60 | SRCi | 2400 |
| 5 | MEKi | 1.5 | BRAFi | 60 | **50** | BRAFi | 60 | STAT3i | 600 |
| 6 | MEKi | 1.5 | PKCi | 3500 | **51** | BRAFi | 60 | mTORi | 0.3 |
| 7 | MEKi | 1.5 | SRCi | 2400 | **52** | PKCi | 3500 |  |  |
| 8 | MEKi | 1.5 | STAT3i | 600 | **53** | PKCi | 3500 | mTORi | 0.3 |
| 9 | MEKi | 1.5 | mTORi | 0.3 | **54** | PKCi | 7000 |  |  |
| 10 | MEKi | 1.5 | PI3Ki | 600 | **55** | CDK4i | 2,000 |  |  |
| 11 | MEKi | 3 |  |  | **56** | CDK4i | 2,000 | MEKi | 1.5 |
| 12 | AKTi | 10,000 |  |  | **57** | CDK4i | 2,000 | AKTi | 5000 |
| 13 | AKTi | 5,000 |  |  | **58** | CDK4i | 2,000 | HDACi | 6000 |
| 14 | AKTi | 5,000 | MEKi | 1.5 | **59** | CDK4i | 2,000 | MDM2i | 3000 |
| 15 | AKTi | 5,000 | HDACi | 6000 | **60** | CDK4i | 2,000 | JAKi | 20 |
| 16 | AKTi | 5,000 | MDM2i | 3000 | **61** | CDK4i | 2,000 | BRAFi | 60 |
| 17 | AKTi | 5,000 | JAKi | 20 | **62** | CDK4i | 2,000 | PKCi | 3500 |
| 18 | AKTi | 5,000 | BRAFi | 60 | **63** | CDK4i | 2,000 | SRCi | 2400 |
| 19 | AKTi | 5,000 | PKCi | 3500 | **64** | CDK4i | 2,000 | STAT3i | 600 |
| 20 | AKTi | 5,000 | SRCi | 2400 | **65** | CDK4i | 2,000 | mTORi | 0.3 |
| 21 | AKTi | 5,000 | STAT3i | 600 | **66** | CDK4i | 2,000 | PI3Ki | 600 |
| 22 | AKTi | 5,000 | mTORi | 0.3 | **67** | SRCi | 2,400 |  |  |
| 23 | AKTi | 5,000 | PI3Ki | 600 | **68** | SRCi | 2,400 | PKCi | 3500 |
| 24 | HDACi | 12,000 |  |  | **69** | SRCi | 2,400 | mTORi | 0.3 |
| 25 | HDACi | 6,000 |  |  | **70** | SRCi | 4,800 |  |  |
| 26 | HDACi | 6,000 | MDM2i | 3000 | **71** | STAT3i | 600 |  |  |
| 27 | HDACi | 6,000 | PKCi | 3500 | **72** | STAT3i | 600 | HDACi | 6000 |
| 28 | HDACi | 6,000 | SRCi | 2400 | **73** | STAT3i | 600 | MDM2i | 3000 |
| 29 | HDACi | 6,000 | mTORi | 0.3 | **74** | STAT3i | 600 | PKCi | 3500 |
| 30 | MDM2i | 3,000 |  |  | **75** | STAT3i | 600 | SRCi | 2400 |
| 31 | MDM2i | 3000 | PKCi | 3500 | **76** | STAT3i | 600 | mTORi | 0.3 |
| 32 | MDM2i | 3000 | SRCi | 2400 | **77** | STAT3i | 1200 |  |  |
| 33 | MDM2i | 3000 | mTORi | 0.3 | **78** | mTORi | 0.3 |  |  |
| 34 | MDM2i | 6,000 |  |  | **79** | mTORi | 0.6 |  |  |
| 35 | JAKi | 20 |  |  | **80** | PI3Ki | 600 |  |  |
| 36 | JAKi | 20 | HDACi | 6000 | **81** | PI3Ki | 600 | HDACi | 6000 |
| 37 | JAKi | 20 | MDM2i | 3000 | **82** | PI3Ki | 600 | MDM2i | 3000 |
| 38 | JAKi | 20 | PKCi | 3500 | **83** | PI3Ki | 600 | JAKi | 20 |
| 39 | JAKi | 20 | SRCi | 2400 | **84** | PI3Ki | 600 | BRAFi | 60 |
| 40 | JAKi | 20 | STAT3i | 600 | **85** | PI3Ki | 600 | PKCi | 3500 |
| 41 | JAKi | 20 | mTORi | 0.3 | **86** | PI3Ki | 600 | SRCi | 2400 |
| 42 | JAKi | 40 |  |  | **87** | PI3Ki | 600 | STAT3i | 600 |
| 43 | BRAFi | 120 |  |  | **88** | PI3Ki | 600 | mTORi | 0.3 |
| 44 | BRAFi | 60 |  |  | **89** | PI3Ki | 1,200 |  |  |

**Table S1D.**Predicted G1-arrest response to combined targeting of c-Myc and other nodes (Perturbations on c-Myc + X). The response values are in log2 and normalized with respect to the response to single agent c-Myc perturbation (G1_c-Myc+X_/G1_c-Myc_). The top 20 paired perturbation conditions are ranked based on the response to the lowest dose (c-Myc_IC20). The _ICX tag determines the strength of the in silico perturbation. The X (e.g, 20, 40) represents the % decrease in the target node activity at the given perturbation strength.

|  | c-Myc_IC20 | c-Myc_IC40 | c-Myc_IC60 | c-Myc_IC80 |
| --- | --- | --- | --- | --- |
| aMEK_IC80 | 2.316 | 1.971 | 1.870 | 1.835 |
| aMEK_IC60 | 2.253 | 1.924 | 1.824 | 1.791 |
| aMEK_IC40 | 2.071 | 1.786 | 1.698 | 1.668 |
| CyclinD1_IC80 | 1.928 | 1.699 | 1.630 | 1.607 |
| CyclinD1_IC60 | 1.893 | 1.670 | 1.606 | 1.581 |
| aBRAFm_IC80 | 1.889 | 1.677 | 1.611 | 1.586 |
| aBRAFm_IC60 | 1.845 | 1.640 | 1.575 | 1.554 |
| CyclinD1_IC40 | 1.795 | 1.594 | 1.536 | 1.515 |
| MAPKpT202_IC80 | 1.725 | 1.535 | 1.478 | 1.458 |
| aBRAFm_IC40 | 1.721 | 1.540 | 1.482 | 1.463 |
| aSRC_IC80 | 1.694 | 1.541 | 1.488 | 1.471 |
| MAPKpT202_IC60 | 1.686 | 1.506 | 1.453 | 1.434 |
| aMEK_IC20 | 1.682 | 1.493 | 1.434 | 1.419 |
| PLK1_IC80 | 1.679 | 1.531 | 1.479 | 1.462 |
| aSRC_IC60 | 1.678 | 1.526 | 1.474 | 1.456 |
| PLK1_IC60 | 1.670 | 1.523 | 1.470 | 1.452 |
| MEKpS217_IC80 | 1.658 | 1.482 | 1.431 | 1.416 |
| PLK1_IC40 | 1.639 | 1.493 | 1.445 | 1.430 |
| aSRC_IC40 | 1.621 | 1.473 | 1.425 | 1.408 |

**Table S1E.** Statistical validation of perturbation models

|  |  | Frequentist coverage probabilities*** | | |
| --- | --- | --- | --- | --- |
| Predictions  (Cross-validation simulations) | **Correlation***  **between the experiments & predictions** | **CI** 95%** | **CI 90%** | **CI 70%** |
| AKTi+Di | 0.81 | 0.96 | 0.93 | 0.77 |
| MEKi+Di | 0.82 | 0.97 | 0.95 | 0.80 |
| CDK4i+Di | 0.69 | 0.84 | 0.73 | 0.48 |
| mTORi+Di | 0.87 | 0.98 | 0.96 | 0.80 |
| RAFi+Di | 0.84 | 0.98 | 0.96 | 0.92 |
| STAT3+Di | 0.80 | 0.97 | 0.95 | 0.86 |
| SRCi+Di | 0.78 | 0.98 | 0.97 | 0.89 |
| PKCi+Di | 0.80 | 0.98 | 0.95 | 0.88 |
| PI3Ki+Di | 0.85 | 0.96 | 0.94 | 0.81 |
| MDM2i+Di | 0.80 | 0.99 | 0.98 | 0.90 |
| JAKi+Di | 0.74 | 0.98 | 0.97 | 0.89 |
| HDACi+Di | 0.74 | 0.93 | 0.88 | 0.75 |

* Pearson’s correlation

**CI: Confidence interval

*** ***Frequentist coverage probability:***

*In nontechnical terms, the frequentist coverage probability is calculated using the distributions of predicted model variables from the simulations of the generated model ensemble with in silico perturbations. In each cross-validation calculation, we first generate a set of models (N=4000 for AKTi+Di and RAFi+Di, N=1000 for all other calculations ) using a partial dataset (Figure3 and Figure S7) and the BP-guided decimation algorithm. Next the models are simulated with in silico perturbations to predict the withheld conditions. The result of the simulations with in silico perturbations is a distribution over all models for each predicted variable (predicted proteomic or phenotypic response). For each predicted distribution, 95%. 90% and 75% confidence intervals are calculated empirically. Next the frequency of true values (i.e., the withheld experimental drug response) that fall in the corresponding confidence intervals is calculated. The resulting frequency gives us the reported frequentist coverage probability for the particular cross-validation calculation and a given confidence interval. The largest confidence interval for which the frequentist coverage probability is higher than the confidence interval value corresponds to the frequentist coverage of the prediction. For all cross-validations except the CDK4+Di and HDACi+Di, the frequentist coverage is higher than 0.95. For HDACi+Di, the frequentist coverage probability is very close to 0.95 for 95% interval, 0.88 for 90% interval and the resulting coverage is >70%.*

***Technical description:*** *For a system with N nodes, an ensemble of model solutions with a size of M (****W_s_****={w_ij_}_s_, ∀i,j, ∈ {1,2,..N}, ∀s ∈ {1,2,..M}) is generated with a BP-guided decimation run (Figure 3 & S7). Simulation of each model in the ensemble with in silico perturbations that target T different nodes (****U****=u_jk_, ∀j,k, ∈ {1,2,..T}) generates a set of predicted model variables (* ***X_P_={x_ijk_}_p_,****∀i ∈ {1,2,..N}* ***,*** *∀j,k ∈ {1,2,..T}****,****∀P ∈ {1,2,..M}) for each inferred model****.*** *As we predict the values of {i} different variables (x_i_) under {j * k} perturbations, we obtain a set of predicted distributions,* ***D =*** *{D_ijk_} over all model solutions. Each* ***x_ijk_*** *is sampled from a distribution, which is a function of model parameters, D_ijk_=F_ijk_(****W****) and has a confidence interval (1-2α) with upper and lower limits, L(x****_ijk_****|****W****) and U(x****_ijk_****|****W****) . The true values of the predicted variables under all perturbation conditions (i.e., hidden experimental response data) is denoted by* ***Y=****{Y_ijk_}****,****∀i ∈ {1,2,..N}, ∀j,k, ∈ {1,2,..T}.* *The frequentist coverage probability of predictions, C_W_^1-2^*^α^ *(****Y****), is defined as the expected probability of* ***Y*** *being within the confidence interval of the predicted distributions,* ***D****:*

*C_W_^1-2^*^α^ *(****Y****)=E****_W_****(P(L_D_ (x_ijk_|****W****) < Y_ijk_ < U_D_(x_ijk_|****W****)))*

*And the frequentist coverage of the predictions is defined as the largest confidence interval, 1-2α, for which the coverage probability is larger than or equal to the confidence level. That is;*

*[E****_W_****(P(L_D_(x_ijk_|****W****) < Y_ijk_ < U_D_(x_ijk_|****W****)))]* ≥ *1-2α*

**Table S1F. Predicted co-targeting strategies in SkMel133 cell line**

| Phenotype | Target1 | Target2 | Response1+2 | Response1 | Response2 |
| --- | --- | --- | --- | --- | --- |
| S-arrest | aCDK4 | STAT3pY705 | 2.02 | 1.71 | 1.46 |
|  | aCDK4 | SMAD3pS423 | 1.88 | 1.71 | 1.26 |
|  | aCDK4 | Fibronectin | 1.83 | 1.71 | 1.12 |
|  | aCDK4 | P27/Kip1 | 1.82 | 1.71 | 1.17 |
|  | aCDK4 | IGF1Rβ | 1.82 | 1.71 | 1.10 |
|  | aCDK4 | TSC2 | 1.81 | 1.71 | 1.19 |
|  | aCDK4 | CRAF | 1.81 | 1.71 | 1.19 |
|  | aCDK4 | BRAF | 1.79 | 1.71 | 1.17 |
|  | aCDK4 | Caveolin | 1.78 | 1.71 | 1.07 |
| G1-arrest | aMEK | cMyc | 1.50 | 1.29 | 1.25 |
|  | aMEK | cJUNpS73 | 1.44 | 1.29 | 1.17 |
|  | aMEK | PLK1 | 1.39 | 1.29 | 1.13 |
|  | aMEK | YB1pS102 | 1.43 | 1.29 | 1.15 |
|  | cMyc | aBRAF | 1.44 | 1.25 | 1.19 |
|  | cMyc | CyclinD1 | 1.43 | 1.25 | 1.19 |
| G2-arrest | aHDAC | STAT3pY705 | 4.61 | 4.50 | 1.08 |
|  | aHDAC | SMAD3 | 4.61 | 4.50 | 1.10 |
|  | aHDAC | TSC2 | 4.61 | 4.50 | 1.07 |
|  | aHDAC | YB1pS102 | 4.61 | 4.50 | 1.07 |
|  | aHDAC | SRC | 4.61 | 4.50 | 1.10 |
|  | aHDAC | CRAF | 4.61 | 4.50 | 1.06 |
|  | aHDAC | RAD51 | 4.61 | 4.50 | 1.06 |
|  | aHDAC | TP53 | 4.61 | 4.50 | 1.10 |
|  | aHDAC | β-cateninpS33 | 4.61 | 4.50 | 1.07 |
|  | aHDAC | SRC | 4.61 | 4.50 | 1.10 |
|  | aHDAC | MAPKpT202 | 4.57 | 4.50 | 1.54 |
|  | aHDAC | Caveolin | 4.57 | 4.50 | 1.08 |
| G2M | aMDM2 | BRAF | 1.70 | 1.52 | 1.15 |
|  | aMDM2 | 4EBP1pT70 | 1.66 | 1.52 | 1.10 |
|  | aMDM2 | ACC1 | 1.58 | 1.52 | 1.06 |
|  | aMDM2 | CRAF | 1.58 | 1.52 | 1.06 |
|  | aMDM2 | TSC2 | 1.58 | 1.52 | 1.06 |
|  | aMDM2 | YAPpS217 | 1.58 | 1.52 | 1.06 |
|  | aMDM2 | GSK3 | 1.57 | 1.52 | 1.05 |
|  | aMDM2 | AKT | 1.57 | 1.52 | 1.05 |
| Cell viability | aPKC | 4EBP1pT70 | 0.09 | 0.16 | 0.33 |
|  | aPKC | P27/Kip1 | 0.09 | 0.16 | 0.36 |
|  | aPKC | P38/MAPK14 | 0.09 | 0.16 | 0.29 |
|  | aPKC | TSC2 | 0.09 | 0.16 | 0.35 |
|  | aPKC | CRAF | 0.09 | 0.16 | 0.31 |
|  | aPKC | ACC1 | 0.09 | 0.16 | 0.42 |
|  | aCDK4 | SMAD3 | 0.09 | 0.16 | 0.30 |
|  | aCDK4 | SMAD3pS323 | 0.09 | 0.16 | 0.33 |
|  | aCDK4 | 4EBP1pT70 | 0.09 | 0.16 | 0.33 |

The top ranked predictions. The letter “a” designates the activity nodes, which couple the effects of the experimental perturbations to the rest of the system. For each phenotype, the top 50 perturbation conditions are listed from the combined list of all perturbations (i.e., perturbations in all 4 strengths) and the quantitative predictions are reported here for the highest perturbation strength for each unique condition. Predictions that involve two activity nodes are excluded from the table. The listed predictions can be tested experimentally using targeted drugs or other methods such as CRISPR/Cas system (Jinek et al, .2012, Cong et al 2013)
